# Life history and nesting traits reflect urban tolerance in coastal birds

**DOI:** 10.1098/rsos.250116

**Published:** 2025-06-25

**Authors:** Sarah L. Jennings, Emma M. Garrison, Clinton D. Francis

**Affiliations:** ^1^ Department of Biological Sciences, California Polytechnic State University San Luis Obispo, San Luis Obispo, CA, USA

**Keywords:** coastal, birds, urban tolerance, functional traits, anthropogenic effects, biodiversity

## Abstract

Rapid urbanization has prompted considerable interest in understanding which species thrive or fail in these novel environments. Because half of the human population resides in coastal areas, studies that explicitly examine urban tolerances among coastal species are needed. Here, we sought to explain variation in coastal bird tolerances to urban habitats with species life history, diet, nest, social, sensory and sexual selection traits using phylogenetically informed models and three urban-tolerance indexes. We found that nest site height was the strongest predictor, with species nesting in elevated locations exhibiting greater urban tolerance, probably due to reduced anthropogenic disturbances and risk of predation. Life-history traits, including larger clutch sizes and lower brood value, reflecting more lifetime breeding attempts, also predicted urban tolerance, suggesting that fast reproductive strategies buffer against urban-associated risks. Contrary to our prediction, species with altricial young displayed higher urban tolerance, potentially due to shorter incubation and fledging times. Collectively, our results suggest that many of the predictors related to urban tolerance in songbirds also predict tolerances among a broader swath of avian diversity. Such knowledge should help researchers forecast the composition of coastal, urban bird communities in the future and will inform efforts to conserve functionally diverse coastal ecosystems.

## 1. Introduction

A pressing concern of the modern world is the impact of urbanization on species and their ecosystems. As of 2018, approximately 55% of the world’s population lives in cities and other urban zones; a number that is projected to increase in the future [1]. Urbanized areas are associated with different resources, sensory stimuli and disturbances from their corresponding natural environments, all of which lead to different ecosystem composition [2,3]. Many taxonomic groups exhibit species-level variation in their ability to tolerate urban environments [4–8]. This interspecific variation in urban tolerance has led researchers to question whether species with certain functional traits (i.e. body mass, diet and life history) that evolved before the spread of urbanization may be better able to exploit and persist in urban areas [9,10]. Such information is critical for conservation experts and policymakers to identify the current and future impacts of urbanization on ecosystems.

Birds are well-suited for examining the relationship between functional traits and urban tolerance [11]. Many birds are easily observed by humans, which has resulted in widespread population data for a large number of species. Additionally, there has been a concerted effort in the ornithological community in recent years to collate and share databases of avian functional traits [12–15]. As with other taxonomic classes, there is notable variation in urban tolerance across birds. Some species are highly tolerant of urban areas [16] where they often exploit anthropogenic resources for diet or nesting [17]. More commonly, many avian species are negatively impacted by the consequences of urbanization, such as the increase of feral and domestic predators [18], greater levels of noise pollution that hinder communication [19] or fatal collisions with reflective surfaces [20].

The majority of studies investigating the relationship between urban tolerance and avian functional traits have included a high proportion of passerine species and thus have provided findings reflective of this group [10,21–23]. The influence of functional traits on urban tolerance in coastal birds is seldom considered in isolation, even though nearly half of the world’s population lives along coastlines [24,25]. Coastal, marine and wetland environments have experienced high levels of degradation [26], with approximately one-third of coastal areas altered by human use and development at the turn of the last century [27]. Given this large and growing influence of human activities on coastal systems, there is increasing concern over declining shorebird and waterbird populations [28–30]. Additionally, marine birds are one of the most endangered groups of birds globally [31,32]. Collectively, these and other coastal-dwelling birds are recognized as important indicators of ecosystem health [33–35], making them an essential group to study in relation to the rapid urbanization of coastal environments.

In the past two decades, an array of studies have proposed indexes to describe the urban tolerance of different species of birds, enlisting various methodologies to do so. A key distinction across these indexes is the use of categorical versus quantitative scoring of urban tolerance. Many initial studies used categorical indexes, assigning species into groups or levels of urban tolerance, such as ‘exploiters and adapters’ [9] and ‘exploiters, avoiders and neutral’ [36]. Subsequent studies sought to develop quantitative indexes where species can take on a wider range of values (e.g. [10,22,37]). These methods offer a key advantage because they capture interspecific variability that can be lost using categorical approaches. Some of the most recently developed quantitative indexes go one step further by quantifying regional differences in species’ urban tolerance across their range [10,38].

The objective of this study is to evaluate which functional traits best predict the ability of coastal bird species to live in urban environments. We define coastal birds broadly as species that rely on coastal environments for habitat and resources. As such, it includes many avian orders, including some passerines. Due to the diversity of existing urban-tolerance indexes, we chose to consider three that vary in methodology, spatial coverage and the diversity of species represented. We used two quantitative measures—the Urban Association Index (UAI) [10] and the Multivariate Urban-Tolerance Index (MUTI) [22]—along with the binary categorical classification of ‘urban’ and ‘non-urban’ proposed by [39]. We modelled the relationship between the three measures of urban tolerance and functional traits from six dimensions: diet, sensory, life history, nest, sexual selection and social traits. These functional traits have either been examined in relation to urban tolerance in previous studies or are novel traits that we hypothesized to be important for urban tolerance in coastal birds (table 1). By focusing exclusively on coastal avian species, we aimed to gain valuable insights into the impact of urbanization on these vulnerable groups and habitats, as well as provide direction for conservation efforts in coastal areas.

**Table 1.** Hypothesized relationships between functional traits and urban tolerance for coastal birds. A predicted negative relationship is represented by ‘−’, a predicted positive relationship by ‘+’ and a prediction of a non-significant relationship as ‘0’. For the three binary categorical variables of developmental mode, nest site height and nest strategy, the levels of each variable are listed. The predicted relationship for categorical traits describes whether the second level of the variables will have higher (+) or lower (−) urban tolerance relative to the first level. The sources for the various traits are noted, and a rationale for each prediction is provided. The final column lists previous studies and their findings.

trait and predicted effect		rationale	prior investigation
body mass [14]	−	food sources used by smaller birds are more readily available in urban areas	positive [37,40] negative [10], no effect [36,39], mixed [41]
*sensory traits*			
dim light vision (this study, [42–44])	−	species with acute dim light vision are more negatively impacted by light pollution in urban areas	no prior investigation
peak vocal frequency [39,45]	0	coastal species have evolved to have vocal frequencies that are audible over low-frequency ocean noise	positive [39,46–48], no effect [49]
*diet traits*			
% invertebrates [12]	−	previous studies have found mixed findings for diet and urban tolerance; as coastal birds exploit different food resources than terrestrial birds, it is relevant to re-examine the relationships between diet and urban tolerance here	negative [9,37,41], no effect [50]
% vertebrates [12]	+		no effect [9,37,51]
% plant/seed [12]	−		positive [9,41,50], negative [16,37,51]
% fruit/nectar [12]	−		no effect [9]
*life-history traits*			
brood value [52,53]	−	species with higher brood values experience greater negative impacts to their lifetime reproductive fitness if their nest fails due to anthropogenic disturbances	negative [36]
clutch size [53]	+	species with higher clutch sizes allocate less energy to each offspring, which have lower energetic costs in urban environments	positive [10,16,37,51,54], negative [55], no effect [9]
longevity [52]	+	longer-lived species are able to acclimate to and learn how to exploit urban environments	positive [10,16,54,55]
developmental mode [56] precocial versus altricial	−	precocial young are better able to tolerate the disturbances of urban environments than altricial young	positive [16], no effect [9,40]
*nest traits*			
nest site height [57] low versus high	+	urbanized areas have fewer suitable ground-nesting sites and increased disturbance to areas the remaining areas avaiable to ground nesting species	positive [10,16,36,41,50,51,58,59], no effect [37]
nest strategy [57] enclosed versus open	_	enclosed nests provide additional protection from pollutants and novel predators compared with open nest	negative [16,36], no effect [10,37]
nest safety [60]	+	nesting strategies that increase safety promote fledgling success in novel urban environments	no prior investigation
*sexual selection traits*			
brightness dichromatism [61]	−	urban areas lack the dietary resources to support strong sexual dichromatism and coloration in urban areas may attract predators	negative [16,59,62]
hue dichromatism [61]	−		
intensity on males [60]	−	species with lower sexual selection intensity (e.g. biparental care) are able to compile reproductive resources and protect young in urban environments.	no prior investigation
intensity on females [60]	−		
*social traits*			
territoriality [63]	_	urban areas do not provide the appropriate habitat features and resources for territorial species to maintain large territories.	negative [10], no effect [9]
cooperative breeding [64]	+	cooperative breeding provides more safety and resources and increases species reproductive success in novel urban environments.	negative [37]

## 2. Methods

### 2.1. Urban-tolerance indexes

We compiled three different indexes from published studies that defined species-specific urban-tolerance values in birds. Two of the three urban-tolerance indexes were quantitative measures that were constructed by pairing high-resolution geospatial data on the degree of urbanization (e.g. nighttime light pollution) with open-access community science data on avian distributions and abundance (e.g. from eBird [65]). The UAI [10] was the most species-rich index with continuous values of urban tolerance for 4415 species from across the globe. The original dataset contains a city-specific UAI score for each species that was calculated by finding the mean light radiance levels for all eBird occurrence records within a city. We averaged the city-specific scores to produce a single UAI score representative of urban tolerance across a species’s entire range. The resulting scores range from 0 to 3.972 with lower values reflecting a greater tendency towards less urban areas. Neate-Clegg *et al*. [10] calculated UAI scores for ‘waterbirds’ but excluded them from their final analyses due to the ecological differences between this group and passerines. As many coastal and marine birds are ‘waterbirds’ and these species are the focus of our analyses, the relationships detected between predictor traits and UAI scores in this study may differ from the findings presented by [10].

We also used a second continuous metric called the MUTI [22] that provides continental-wide measures of urban tolerance for 432 species from North America. MUTI is based on two different calculations of urban tolerance; the first equation quantifies how abundant species are, on average, in urban areas relative to non-urban areas, and the second measures the intensity of nighttime light pollution in the areas each species uses relative to areas within its range that were unused. MUTI scores are the first principal component from a principal component analysis using these two calculations, which explained approximately 93% of the variation in urban tolerance across species. Species with higher MUTI values reflect greater urban tolerance; scores range from −2.049 to 5.461.

Finally, we used the binary classification of non-urban (N) and urban (U) that contains values for 529 species from Europe, North America and Australia (hereafter UN [39]). UN represents one of the earlier approaches to quantifying urban tolerance; it uses species habitat descriptions to determine whether each species uses urban areas or not.

We selected these three indexes because they represent a range of approaches for assigning urban tolerance to species, and they encompass a variety of geographic areas and thus include different species. The number of species with values across all three indexes was low (*n* = 180), and the correlations between the indexes for the shared species were moderate to weak (electronic supplementary material, figure S1). As such, we did not necessarily expect them to produce the same insights. However, we considered traits with relationships to urban tolerance across more than one index as strong support for that trait being globally important for the ability of coastal birds to persist in urban areas.

All indexes were combined into a database so that each row contained an individual species and their urban-tolerance scores from one or more of the three indexes, an English common name, their taxonomic Family and scientific names from the three widely used nomenclature schemes: BirdLife International [66], BirdTree [67] and eBird [68]. After joining, our list contained 4433 total unique species, of which, 4347 species have UAI scores, 528 species have UN scores and 431 species have MUTI scores.

### 2.2. Identifying coastal species with urban-tolerance scores

**Figure 1.**
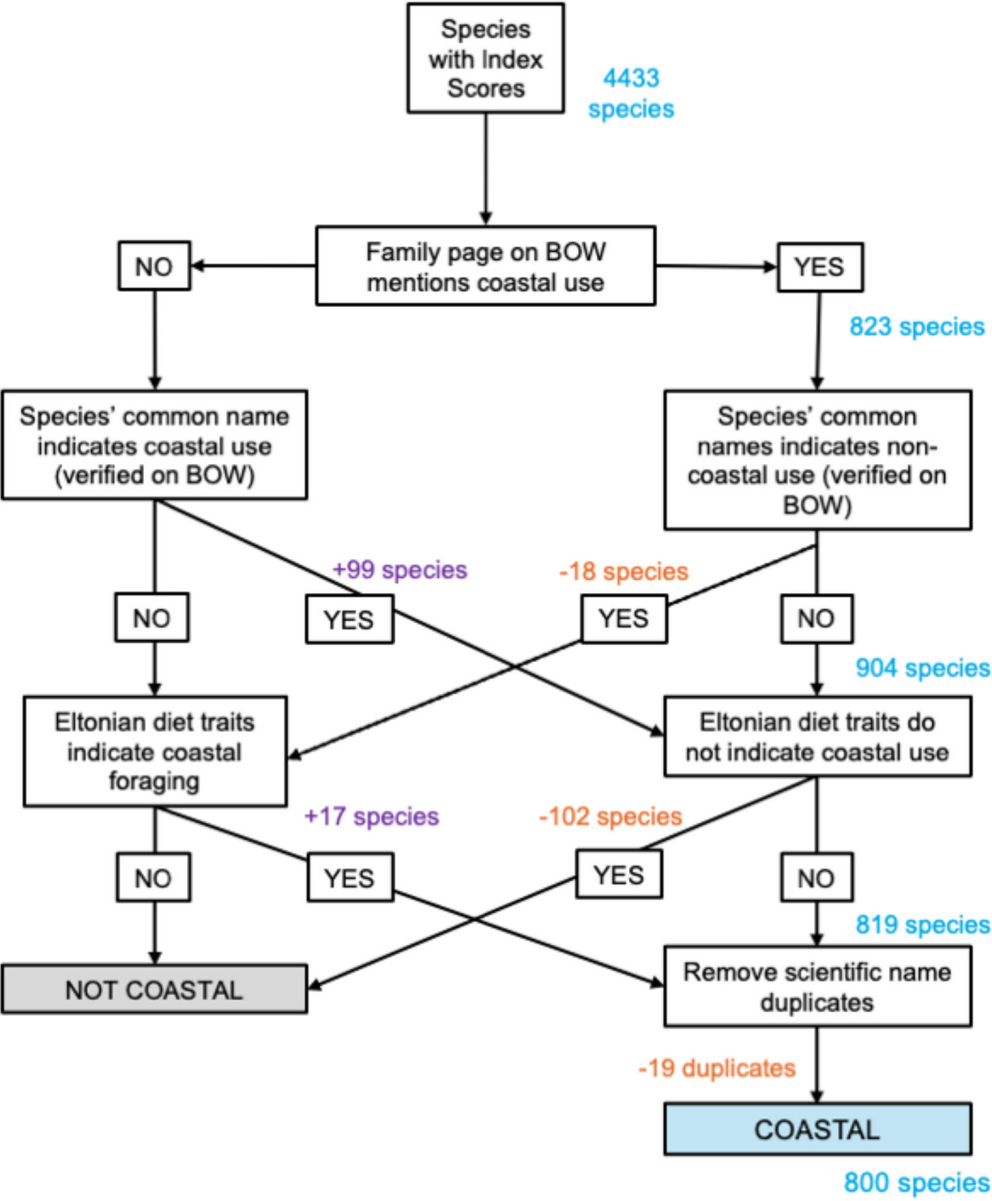
Methodology for refining avian species down to a list of species that rely on coastal environments. Blue numbers represent the total number of coastal species after each step of the filtering process. Orange numbers reflect the number of species removed from the coastal list, and purple numbers represent the number of species added to the coastal list. BOW, Birds of the World database [69].

We used the list of 4433 species that had at least one urban-tolerance score and performed a multi-step filtering process to produce a list of coastal bird species (figure 1). We defined a coastal bird as a species that spends a significant portion of their annual cycle using resources and occupying habitats encompassed by oceans and seas, or where tides are integral; this includes shorelines, beaches, estuaries, mangroves, tidal flats and marine environments. Our filtering process aimed to capture all index-represented birds that use coastal habitats and resources. However, we did not perform an exhaustive search of all 4433 species, so our final species list may have missed some species that interact with coastal environments.

For the first round of filtering, we aligned the 4433 species to their taxonomic family (*n* = 205). All families were investigated via the family accounts in the Cornell Laboratory of Ornithology Birds of the World database (hereafter BOW; [69]) for any mention of coastal habitat or resource use. Each family, and thus all representative species within the family, was classified as either ‘coastal’ or ‘non-coastal’. This produced a list where 37 families containing 823 species were marked as ‘coastal’ and 168 families containing 3610 species were marked as ‘non-coastal’. In the subsequent rounds of filtering, birds were evaluated at the species level and were able to move between the coastal and non-coastal classification groups.

The second round of filtering further evaluated the species belonging to ‘coastal’ families for outlier species that should actually be classified as ‘non-coastal’ birds. To do so, we looked to species’ common names for clues. We generated a list of all descriptor terms seen in the common names of our original 4433 species list. Two researchers independently identified all terms in this list that related to diet, habitat or an association between the bird and another species and manually assigned a coastal association category to each term. Words such as highland, mountain, alpine were marked as ‘not coastal’ (N); words such as bank, island, palm were marked as ‘maybe coastal’ (M); and words such as surf, beach, kelp were marked as ‘yes coastal’ (Y). We cross-checked the descriptive terms against the classifications made in round one and identified 39 species marked as ‘coastal’ that contained common names with ‘not coastal’ (N) descriptor terms. Each of these species was manually inspected on BOW [69] for mention of coastal use, resulting in 18 species being reassigned as ‘non-coastal’.

For the third round of filtering, we conducted the inverse of round 2, using common name descriptor terms to examine the species initially categorized as ‘non-coastal’ for outliers that may use coastal habitats. Using the previously compiled list of common name habitat/diet descriptive terms, we identified 221 species within the list with ‘maybe coastal’ (M) and ‘yes coastal’ (Y) terms in their common names. After consulting BOW species accounts [69], we reassigned 99 species as ‘Coastal’.

The fourth round of filtering steps incorporated the Eltonian diet and foraging behaviour traits from [12]. We considered the following traits as indicative of species that may forage in coastal areas or on coastal resources: (i) a non-zero percentage of fish in its diet; (ii) forages at the water’s surface; (iii) forages below the water’s surface; (iv) is classified as a ‘pelagic specialist’. We examined all species marked as ‘non-coastal’ based on the four diet and foraging traits and reclassified 17 species as ‘coastal’ after consulting the species accounts for each on BOW [69].

For the fifth round of filtering, we applied the inverse of round 4 to evaluate species previously assigned as ‘coastal’ that do not possess coastal diet and foraging traits. After reading the BOW species accounts [69], we reclassified 102 species as ‘non-coastal’. At this stage, 819 species were on the coastal birds list.

As a final step, we checked that all the species we had classified as coastal had a unique scientific name using the BirdTree taxonomy [67], which was employed for the subsequent phylogenetic trait-based analysis. There were 36 species with duplicated scientific names, which was expected as the other naming schemes (BirdLife [66] and eBird [68]) in our dataset contain more unique species names than the BirdTree taxonomy. We manually investigated these species to resolve duplicate names, retaining 17 and dropping 19 of the 36 species. We were left with 800 coastal birds. This list represents avian species across the world that rely on coastal habitats for feeding and/or breeding that have urban-tolerance scores in at least one of the three datasets (UAI, UN or MUTI).

### 2.3. Urban-tolerance context and phylogenetic representation

After filtering each index to contain only coastal birds, UAI had 791 species, while MUTI and UN both had 128 species. To provide context for how urban tolerances among the coastal birds in our dataset compared to urban tolerances of birds in general, we compared the mean urban-tolerance score of the subset of coastal species with the mean score of the complete species list for MUTI and UAI. We performed a similar comparison for the UN index by calculating the percentage of birds classified as Urban and Non-Urban within the coastal species and all species list. To get a better sense of how the three indexes differed in the types of coastal birds they represent, we examined the phylogenetic diversity of the three indexes by calculating phylogenetic species variability (PSV) and phylogenetic species richness (PSR) from [70] using the phyr package in R [71,72]. PSV quantifies how phylogenetic relatedness decreases the variability of traits within a group, in this case, a list of species represented by a specific urban-tolerance index. This value is bounded by 0 and 1 with higher values indicating more diversity and fewer close relatives. PSR is the species richness multiplied by the PSV and is directly related to actual species richness. PSR values much lower than species richness indicate that there are many close relatives within the group.

### 2.4. Species trait data

We compiled species trait data from various sources and linked them with the three measures of urban tolerance (UAI, UN and MUTI). Many of these trait data have been previously identified as important by other studies focused on urban tolerance in birds (table 1). In total, our traits encompass six broad categories that we hypothesized were related to urban tolerance: sensory, diet, life history, nesting, sexual selection and social traits. The number and exact species represented by each trait dataset varied, and accordingly, the number of species within each urban-tolerance index where trait information was available also differed (see electronic supplementary material, table S1 for sample sizes). Below, we provide a rationale for the inclusion of each trait group and a brief description of individual traits. Trait-related hypotheses and predictions for each model are provided in table 1.

#### 2.4.1. Sensory traits

Urban environments contain novel sensory stimuli including chemicals, light and noise from anthropogenic sources. Altered sensory landscapes can mask an organism’s ability to detect signals and cues, may distract organisms and can mislead organisms leading to maladaptive behaviours [73]. In birds, anthropogenic noise and light pollution can impact nest success and phenology [74]; thus, interspecific variation in avian vocal and visual traits may be relevant when considering urban tolerance.

We obtained peak vocal frequency (Hz) from [45] and [39]. We used the ratio of the width of the cornea to the width of the transverse of the eye orbit (hereafter CT ratio) as a proxy for dim light vision [42,74,75]. We either collected these data from museum specimens (*n* = 23) or obtained these data from several different sources [42–44].

#### 2.4.2. Diet traits

Urbanized areas provide different dietary resources than adjacent natural areas, with an influx of supplemental feeding opportunities from anthropogenic sources [76,77] and greater fragmentation of native vegetation and nutrient cycles [78]. Many previous studies have evaluated whether the composition of species’ diets impacts their ability to thrive in urban areas [9,37,40]. We examined four different diet categories to determine whether urban tolerance is related to the degree that a species relies upon a particular food source. We obtained diet data for all species from [12]. The 10 original diet categories in this dataset were combined and reduced into four categories that reflected the percentage of diet composed of (i) vertebrates (endotherms, ectotherms, fish, scavenging and unknown vertebrates); (ii) invertebrates (which includes both aquatic and terrestrial invertebrate lineages); (iii) fruit and nectar; and (iv) plants and seeds.

#### 2.4.3. Life-history traits

Life-history traits reflect the variation in breeding strategies among birds and may affect a species’ ability to exploit and colonize areas with environmental uncertainties [16,54]. Because urbanized areas may present a variety of environmental factors not seen in natural areas, life-history traits should be considered to determine if there is a different optimal life-history strategy in urban areas compared to natural ones [55]. We focused on four different life-history traits.

We used the trait brood value [36,79] to quantify the relative significance of each nesting attempt across species. Species that have many breeding attempts over their lifetime have a lower brood value, and species with few lifetime breeding attempts have a higher brood value. Brood value was calculated as log(1/total no. of lifetime breeding attempts) where the number of lifetime breeding attempts was equal to the lifespan of each species multiplied by the number of clutches they lay each year. We used maximum lifespan values from [52] and obtained the number of nesting attempts per year from [53]. We identified and modified one outlier value for killdeer (*Charadrius vociferus*), where the number of clutches per year from [53] was listed as 15. After consulting BOW, we adjusted the clutches per year for this species to 1.

We also obtained categorical developmental mode data (0 = precocial and 1 = altricial) from [56]; longevity data (maximum lifespan in years) from [52]; and clutch size data (number of eggs per clutch) from [53].

#### 2.4.4. Nest traits

Within urban environments, threats towards nests, such as direct anthropogenic disturbance or urban predator populations, may differ from adjacent natural environments [80]. Additionally, the availability of suitable nesting substrates is different between the urban and natural environments [81]. We used three nesting traits to examine whether nesting location, architecture and safety are related to a species’ tolerance for urban environments.

We obtained nest trait data from [57], who describe seven nest site categories. We binned these seven nest site categories into two categories: high nest sites (tree, non-tree and cliff or bank) and low nest sites (ground, underground and waterbody). Many coastal birds are flexible in their nesting location and occupy both low and high nest sites (222 out of 800 species). For example, the black oystercatcher (*Haematopus bachmani*) often breeds on shorelines where individual nests may be located either on the ground or on cliffs [82]. Because of the prevalence of flexible nesting sites in our dataset, we retained three binary nest site groupings as predictor traits. The first grouping parsed species that always or occasionally use high nests into one category and left all species that exclusively use low nest sites in the other. In contrast, the second grouping combined all species that always or occasionally use low nest sites into one category and put species that exclusively use high nest sites into the other category. A third and final grouping removed all species with flexible nesting locations and retained species that only use high or low nest sites as the two categories. We also obtained information on nesting strategy from [57]. We binned seven nesting strategies into two categories that represent open (scrape, platform and cup) and enclosed (simple dome, dome with tunnel, primary cavity and secondary cavity) nest structures. There were 56 species that were marked as using both open and closed nests. We investigated the predominant nesting strategy for these species on BOW and assigned 32 species to a single primary strategy. The 24 species that could not be clearly resolved were dropped from the analysis. Additionally, we obtained nest safety values from [60], which is a metric created by adding nest location scores and nest content visibility scores (open or enclosed). Nest safety ranges from 0 to 4, representing a gradient of low to high nest safety.

#### 2.4.5. Sexual selection traits

Few studies have examined the role of sexual selection in predicting species’ urban tolerance, and there are currently mixed predictions about whether strongly sexually selected species will thrive or struggle in anthropogenically altered environments [62,83,84]. Urban areas provide dramatically different landscapes, altered food resources and increased pollution that can change the vulnerability of birds to predators (e.g. making them either easier or harder to detect) and can impact their ability to communicate with conspecifics (e.g. improved or reduced signalling; [85]). We investigated how sexual selection relates to urban tolerance in coastal birds using the degree of intraspecific colour dimorphism between males and females, which is one proxy for the strength of sexual selection [61,86,87] that may be related to both camouflage from predators and sexual communication with conspecifics. We examined two measures of plumage dichromatism from [61]: the degree of sexual dichromatism of plumage brightness and the degree of sexual dichromatism of plumage hue. We took the absolute value of the dichromatism scores for both brightness and hue. After making this transformation, larger values represented higher levels of plumage sexual dichromatism, and values closer to 0 represented more sexually monochromatic species.

Whether plumage dichromatism is always an accurate measure of sexual selection intensity in birds has been questioned, as sexual selection may act on non-visual traits [88] and natural selection can also shape plumage coloration [61]. Therefore, we obtained two additional metrics of sexual selection that are based on mating systems from [60], which included the intensity of sexual selection on males and likewise on females. This index is derived from social mating and parental care systems and ranges from 0 (monogamy and biparental care) to 5 (polygyny/polyandry and uniparental care).

#### 2.4.6. Social traits

The resources in urban environments may not be able to support highly territorial species or oppositely may promote the success of highly territorial/aggressive species [89]. Additionally, social species that are cooperative breeders may have the behavioural flexibility to tolerate the ecological pressures of an urbanized environment [90]. Therefore, the social traits of avian species may influence their likelihood to exploit urban areas.

We collected cooperative breeding trait data from [64], where species were classified as yes (cooperative breeding) or no (non-cooperative breeding). Territoriality data were collected from [63], which classified species under three levels: 0 = non-territorial, 1 = weakly or seasonally territorial and 2 = year-round territorial.

### 2.5. Modelling the predictor traits with the indexes for urban tolerance

We used R (v4.3.2) statistical software [91] implemented in RStudio (v2024.04.2+764) [92] to conduct all statistical analyses. We fit phylogenetic linear (phylogenetic generalized least squares, ‘PGLS’) and logistic models that predicted the relationship between species’ urban tolerance and the 21 predictor traits while controlling for relatedness among species using the phylogeny of all extant birds [67]. Because sample sizes varied for each trait, each model contained a trait (i.e. peak vocal frequency, brood value, etc.) as a fixed effect and one of the three urban-tolerance indexes (UAI, MUTI or UN) as the response variable (electronic supplementary material, table S1). To account for the potential relationships between body mass and other predictor traits, we included log-transformed body mass as a covariate in all models (body mass values from [14]). In addition to the models described above, we considered body mass independently as a predictor variable to explain urban-tolerance in coastal birds. Some discrete predictor traits had categories with fewer than 10 species (electronic supplementary material, table S1). Due to the statistical unreliability of small sample sizes, we excluded these models from our results.

All PGLS models for the continuous urban indices of UAI and MUTI were fitted using the *gls* function in the package nlme [93,94]. For the categorical UN index, we used the *phyloglm* function with the logistic_MPLE method from the package phylolm [95], which implemented a phylogenetic logistic model suitable for a binary response variable [96].

To quantify phylogenetic signals between a trait and an urban-tolerance index, we estimated the phylogenetic correlation parameters ‘Pagel’s lambda’ (*λ*) for PGLS models and ‘alpha’ (*α*) for logistic models. Lambda (*λ*) takes on values from 0 to 1, with larger values reflecting stronger phylogenetic signals and thus greater support for a Brownian motion model of trait evolution. For models that failed to converge, we explored fixed values of *λ* in intervals of 0.1 from 0 to 1 and recorded the corresponding Akaike’s information criterion (AIC) score for each model. The *λ* value with the lowest AIC score was selected and set as a fixed value in the final model. In contrast, *α* ranges from 0 to ∞ where *α* values near or equal to zero reflect greater support for a strong phylogenetic signal. For practical purposes, the *phyloglm* function sets an upper bound on *α* of *e*^4^/*t* where *t* is the mean tip height of the phylogenetic tree; the regression coefficients at this boundary should be similar to a non-phylogenetic logistic regression model with Firth’s correction [96]. When models failed to converge on a value of *α*, we implemented a similar process of inspecting the AIC scores of a series of models where the upper boundary for the log of *α* increased in intervals of 0.1 up the default max value of 4. The *phyloglm* function does not allow the user to specify fixed values of *α*; instead, we achieved model convergence by restricting the bounds of *α* to a narrow search range that encompassed the *α* value that produced the lowest AIC score. If one of our phylogenetic logistic models converged at the upper boundary for *α*, reflecting little support for a phylogenetic signal in the model, we also ran a logistic regression using Firth’s correction using the *logistf* function in the logistf package [97] and compared the estimated coefficients to ensure that both approaches led to the same conclusions.

For each PGLS model, we performed diagnostics using the performance package [98], which were manually inspected to confirm fit to model assumptions. Visual model diagnostics were unavailable for phylogenetic logistic regression models, so we estimated robust model parameters using 1000 independent bootstrap replicates of the data.

We report effects if the 95% confidence interval (95% CI) for the parameter estimate did not overlap zero. Because we embrace recommendations from statisticians to use a more nuanced approach to reporting parameter estimates than dichotomous significance testing [99–102], we also present and discuss models with notable trends where the 95% CIs slightly overlap zero. We visualized relationships and trends between predictor traits and urban-tolerance metrics using the ggplot2 package [103].

## 3. Results

**Figure 2.**
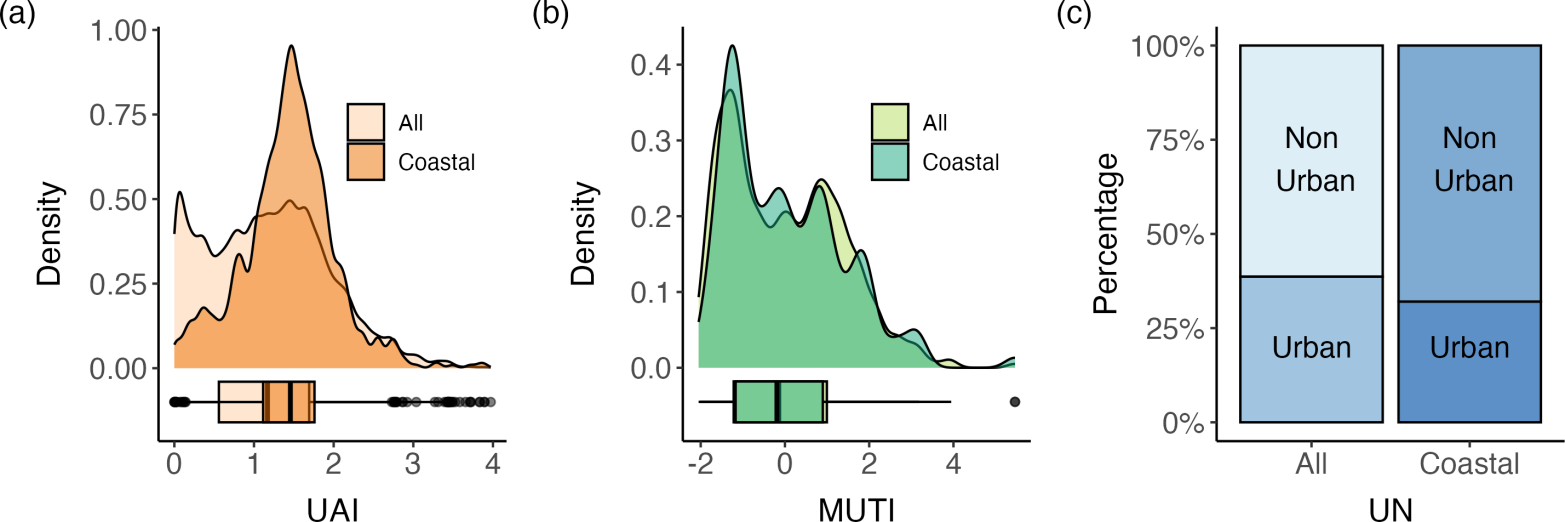
Distributions of urban-tolerance scores for (a) UAI, (b) MUTI and (c) UN indexes. The distribution of scores for coastal birds (darker) is overlaid on top of the distribution of scores for all birds contained in the index (lighter) for UAI and MUTI. The percentage of urban birds for all birds and the coastal species are shown side by side in a stacked, percentage bar chart for UN.

When using UAI to quantify urban tolerance, we observed that the subset of coastal birds has a higher average urban tolerance than all bird species combined (mean UAI for coastal species = 1.433, mean UAI for all birds = 1.182), a finding that was due to species with low UAI scores between 0 and 1 being less common in coastal birds compared with the complete list of species in the UAI dataset (figure 2a). This pattern was not evident when considering MUTI or UN where the distributions of urban-tolerance scores for all bird species were similar to the distribution of scores for coastal-only species. The average MUTI value for coastal species was close to zero (mean MUTI for coastal species = 0.0176, mean MUTI for all species −0.0051; figure 2b), which corresponds with neutral urban tolerance. For UN, the majority of coastal birds were classified as ‘non-urban’, as was the case for the list of all bird species (coastal species = 68%, all species = 61%; figure 2c).

**Figure 3.**
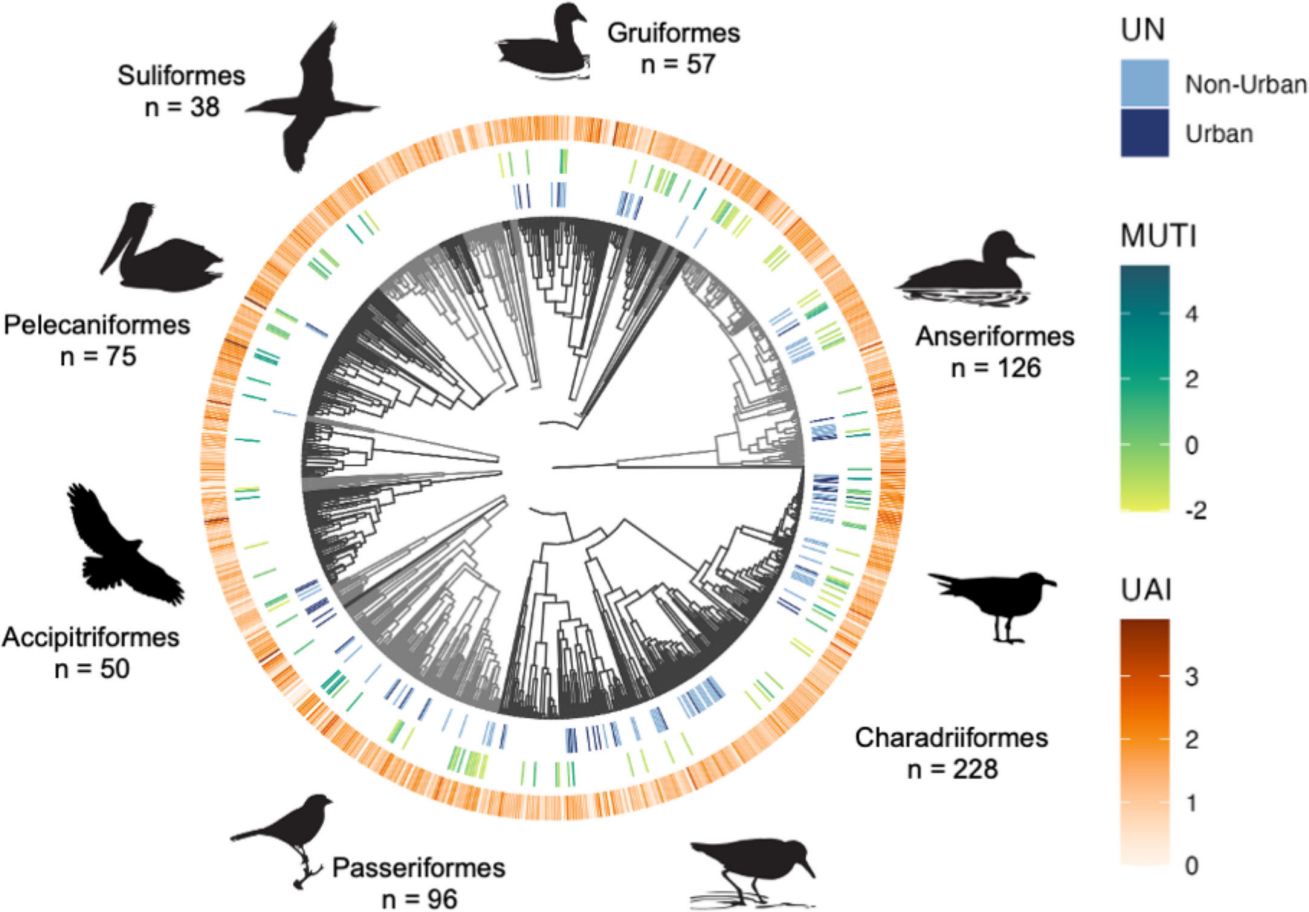
Phylogeny of coastal bird species with species-specific urban-tolerance scores for the three indexes depicted in the outer rings. Phylogenetic tree is shaded to show the 23 avian orders represented in the dataset. Orders with 30 or more species are labelled with representative silhouettes and the number of species. Silhouettes from All About Birds [104].

Unsurprisingly, UAI with the largest number of coastal species (*n* = 791) also represented the most avian families (*n* = 81; table 2). MUTI and UN contained the same number of coastal species (*n* = 128), but MUTI included more families than UN (32 versus 24; table 2). All three indexes had similar levels of PSV with values close to 1 reflecting the high taxonomic diversity represented by the species within each index (table 2 and figure 3). PSR was slightly lower than actual species richness for all indexes, probably due to the presence of some closely related species (table 2 and figure 3). The number of coastal species with urban-tolerance scores for all three indexes was only 49, so while each index captured a high level of phylogenetic diversity, they differed substantially in the species and families they represented (figure 3). See electronic supplementary material, table S3, for the most and least urban-tolerant coastal species based on the UAI and MUTI indexes.

**Table 2.** Phylogenetic diversity measures for the three urban-tolerance indexes (UAI, UN and MUTI).

urban-tolerance index	no. of families	species richness	PSR	PSV
UAI	81	791	637	0.804
MUTI	32	128	102	0.797
UN	24	128	97	0.760

**Figure 4.**
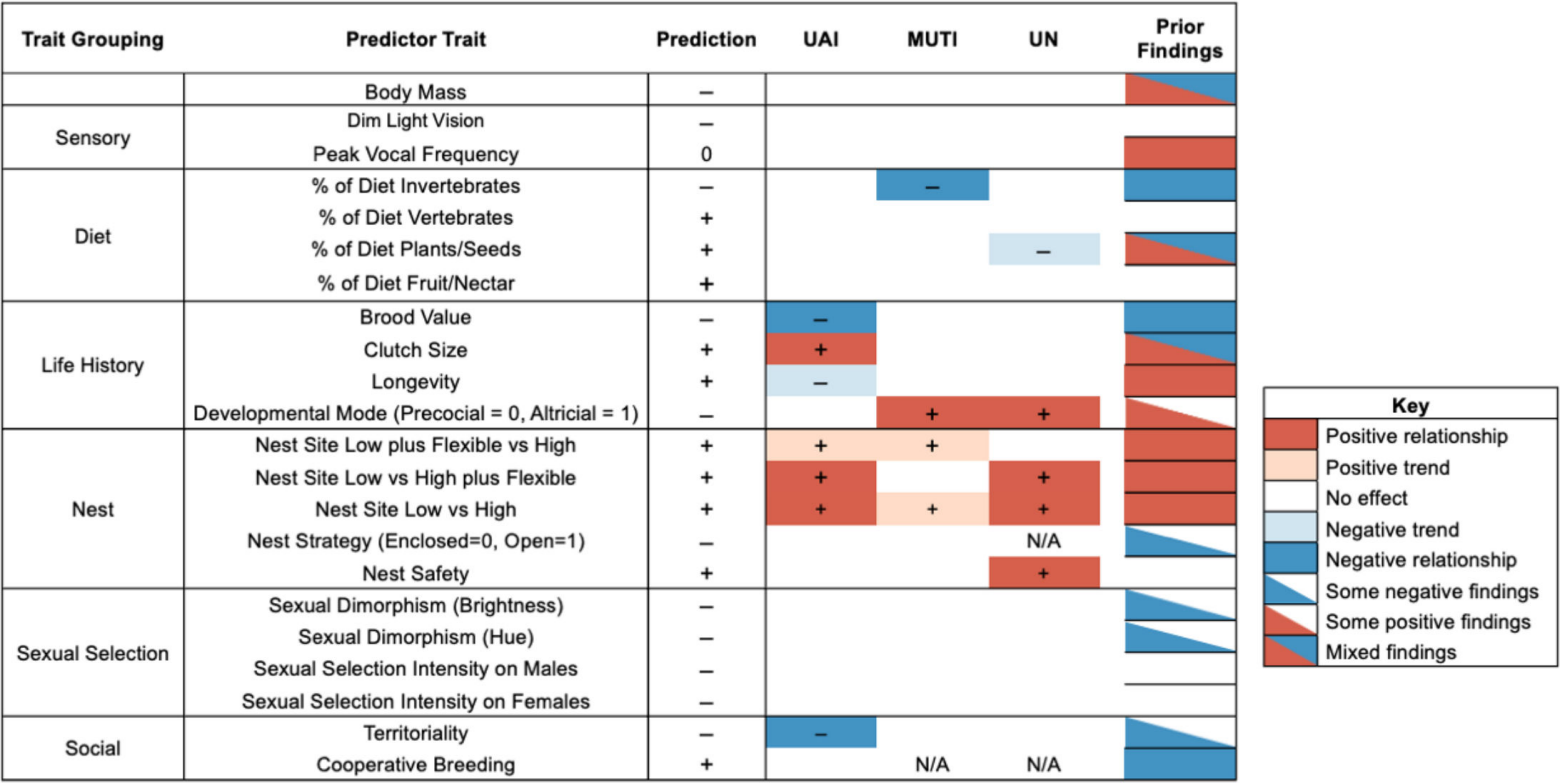
Results for all trait models examining the relationship between ecological traits and measures of urban tolerance. Predicted relationships (from table 1) for each model are noted to the left of model results. Models that found negative relationships between a trait and urban tolerance are represented by (−), and positive relationships are represented by (+). Darker shading denotes models where the 95% CI for the trait did not overlap zero, while lighter shades reflect models with trends where the 95% CI slightly overlapped zero. ‘N/A’ identifies models that could not be run, due to insufficient sample sizes.

The urban tolerance of coastal birds was best explained by nesting traits and life-history traits (figure 4; electronic supplementary material, table S2). There were some relationships between urban tolerance and diet traits and one finding for social traits (figure 4; electronic supplementary material, table S2). We found limited support for a relationship between urban tolerance in coastal birds and body mass, sensory traits or sexual selection traits. For predictor trait categories that were found to have relationships or trends with the urban tolerance of coastal birds, further information is provided below.

### 3.1. Diet traits

We identified two diet traits that were related to urban tolerance in coastal birds. Species with a higher proportion of invertebrates in their diets have lower urban tolerance as described by the MUTI index (*β* = −0.012, s.e. = 0.005, 95% CI = −0.021, −0.002, *λ* = 0.245; electronic supplementary material, table S2). Species that consume a high proportion of plants and seeds tended to be ‘non-urban’ according to the UN index, although the precision of the estimated effect for this relationship was lower as the 95% CI slightly overlapped zero (*β* = −0.509, s.e. = 0.270, 95% CI = −1.037, −0.020, *α* = 0.106; electronic supplementary material, table S2). We found no relationships between diet traits and urban tolerance when using UAI as the measure of urban tolerance.

### 3.2. Life-history traits

**Figure 5.**
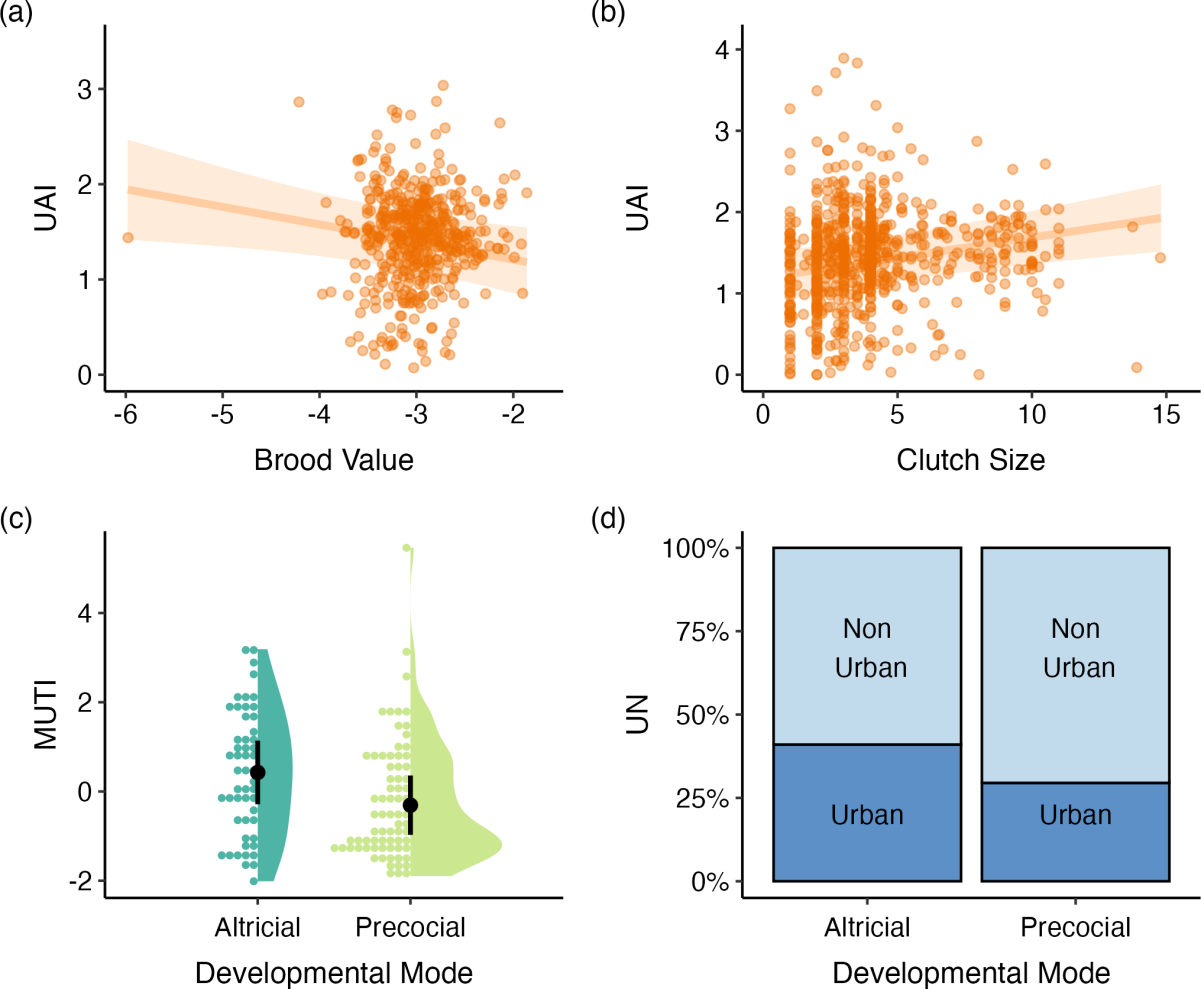
Life-history traits predict urban tolerance in coastal birds. (a) Species with lower brood values and (b) larger clutch sizes exhibit higher urban tolerance using UAI. Species with altricial developmental modes are more urban tolerant than precocial species when using the (c) MUTI and (d) UN indexes. Each panel depicts the predicted marginal effect of the focal trait, which is shown as a trend line with 95% CI (a,b), a mean and 95% CI represented by the black circle and bars (c) or as percentages of urban and non-urban species (d). Raw data for the focal traits are depicted by points (a,b) and using dot and violin plots (c).

Several life-history traits showed strong relationships with urban tolerance in coastal birds. Species with higher brood values that have fewer breeding attempts over their lifetime have lower urban tolerance when using the UAI index (*β* = −0.184, s.e. = 0.077, 95% CI = −0.335, −0.034, *λ* = 0.450; figure 5a; electronic supplementary material, table S2). Removing the Australian brushturkey (*Alectura lathami*) from this model, a polyandrous species with an extreme brood value of −5.98 does not impact this finding (*β* = −0.213, s.e. = 0.083, 95% CI = −0.377, −0.050, *λ* = 0.455). We also found that clutch size was positively related to urban tolerance for coastal species when using the UAI index (*β* = 0.050, s.e. = 0.015, 95% CI = 0.021, 0.078, *λ* = 0.316; figure 5b; electronic supplementary material, table S2). Moreover, urban tolerance for coastal birds is higher in species with altricial young than precocial young: a finding that was supported by MUTI (*β* = 0.871, s.e. = 0.375, 95% CI = 0.136, 1.606, *λ* = 0.201; figure 5c; electronic supplementary material, table S2) and UN (*β* = 1.009, s.e. = 0.485, 95% CI = 0.058, 1.961, *α* = 0.558; figure 5d; electronic supplementary material, table S2). Additionally, we found a trend that suggests that longer-lived birds may have lower urban tolerance for UAI (UAI: *β* = −0.008, s.e. = 0.006, 95% C.I. = −0.020, 0.003, *λ* = 0.309; electronic supplementary material, table S2).

### 3.3. Nest traits

**Figure 6.**
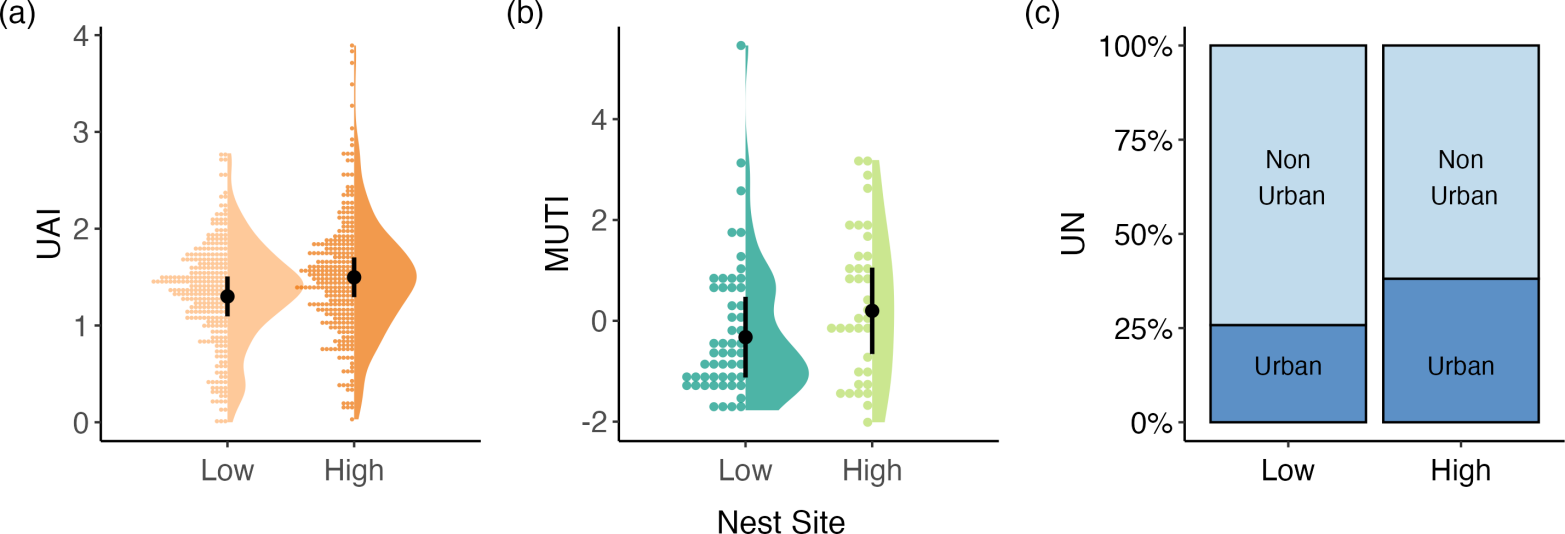
Off-ground nest sites in high locations were associated with increased urban tolerance for coastal birds across all three indexes: (a) UAI, (b) MUTI and (c) UN. The two groups in each panel represent species that exclusively nest on the ground (left) and species that exclusively use elevated nest sites (right). Each panel depicts the predicted marginal effect of the focal trait, which is shown as a mean and 95% CI represented by the black circle and bars (a,b) or as percentages of urban and non-urban species (c). Raw data for the focal traits are depicted by using dot and violin plots (a,b).

Coastal species that utilize nests that are elevated off the ground are more urban tolerant than species that nest close to the ground. All three indexes exhibited a positive relationship between high nest sites and urban tolerance when flexible species that can occupy both low and high nest sites (e.g. ground and cliffs) were removed from the analysis (UAI: *β* = 0.188, s.e. = 0.069, 95% C.I. = 0.054, 0.323, *λ* = 0.148; MUTI: *β* = 0.611, s.e. = 0.370, 95% CI = −0.114, 1.335, *λ* = 0.300; UN: *β* = 1.126, s.e. = 0.537, 95% CI = 0.074, 2.178, *α* = 0.559; figure 6; electronic supplementary material, table S2). The relationships for UAI and UN had 95% CIs that did not overlap zero, while the precision of the estimated effect for MUTI was lower because the 95% CI slightly overlapped zero. When flexible species were included in the analysis and binned with those that use high nest sites, we found this group had higher urban tolerance than species that exclusively use low nest sites for both UAI and UN (UAI: *β* = 0.159, s.e. = 0.051, 95% CI = 0.059, 0259, *λ* = 0.352; UN: *β* = 0.928, s.e. = 0.408, 95% CI = 0.127, 1.728, *α* = 0.558; electronic supplementary material, table S2). In contrast, when the flexible species were grouped with the low nesting species, we found trends to suggest that species that exclusively high nest sites may have higher urban tolerance for MUTI and UAI (MUTI: *β* = 0.443, s.e. = 0.298, 95% CI = −0.141, 1.027, *λ* = 0.157; UAI: *β* = 0.098, s.e. = 0.056, 95% CI = −0.011, 0.208, *λ* = 0.348; electronic supplementary material, table S2). Additionally, coastal species with safer nests (higher locations and less visible) had a higher probability of being classified as ‘urban’ using the UN index (*β* = 0.473, s.e. = 0.207, 95% CI = 0.067, 0.880, *α* = 0.557; electronic supplementary material, table S2). We found no evidence that the nest structure of coastal species (whether they rear young in open or enclosed nests) is related to urban tolerance across any of the three metrics.

### 3.4. Social traits

Territoriality was negatively related to urban tolerance in coastal birds when using UAI (*β* = −0.092, s.e. = 0.047, 95% CI = −0.183, 0.000, *λ* = 0.375; electronic supplementary material, table S2). Pairwise comparisons of the differences between the three levels of territoriality revealed that non-territorial species had higher urban tolerance than weakly or seasonally territorial species (contrast = 0.14, 95% CI = 0.01, 0.26), and there was a trend to suggest that non-territorial species may be more urban tolerant than species that exhibit year-round territoriality (contrast = 0.16, 95% CI = 0.03, 0.35). No other social traits had relationships with urban tolerance.

## 4. Discussion

Several previous studies have examined how species-specific functional traits are related to urban tolerance in birds with a focus on passerines (order Passeriformes), but none have examined urban tolerance specifically in coastal birds. Due to the rapid urbanization of coastlines [105,106], the threatened or endangered status of many shorebirds and seabirds [30,32] and the broad range of ecological traits present across birds that occupy coastal areas [107–109], it is important to consider how birds that rely on these habitats may respond to ongoing urbanization. Accordingly, we evaluated whether the urban tolerance of coastal birds can be explained by ecological functional traits using three previously developed indexes of urban tolerance. Because our focus was on species that inhabit shorelines, estuaries, mangroves and nearshore marine habitats, our species list encompasses a wide range of avian orders (*n* = 23). In contrast with previous studies, only 12% of the species in our analyses were passerines. We found that coastal species that lay larger clutches, produce altricial young, have more reproductive events across their lifetime (low brood value) and nest in elevated locations exhibit higher urban tolerance. They are also likely to be non-territorial and have diets that contain a low percentage of invertebrate prey.

Nest site height was the most consistent predictor of urban tolerance in coastal birds, with support found across all three indexes when excluding species with flexible nest sites. Our results indicate that coastal bird species that use nest sites in elevated locations have greater urban tolerance than those nesting on the ground. Off-ground nest sites such as trees, buildings and cliffs may be less prone to anthropogenic disturbances [110], allowing birds that use these locations to be more ‘urban tolerant’. Previous studies have shown that ground-nesting coastal birds can be sensitive to human disturbances, but species often vary in their sensitivity [111–113]. Abandoning their nest, even for short periods of time, can have high fitness consequences; therefore, further research to identify which ground-nesting coastal species are most sensitive to anthropogenic disturbances is needed to inform conservation practices such as limiting human access to the areas where vulnerable species are breeding [114,115]. Additionally, non-native mammalian predators represent a major threat to shorebirds and seabirds, and species that nest on the ground are particularly vulnerable [116,117]. Efforts to remove non-native predators from islands are costly but often effective for protecting breeding birds. For example, predator removal has restored population growth rates for Cook’s petrels (*Pterodroma cookii*) and Sooty terns (*Onychoprion fuscatus*), two species with low urban-tolerance scores for UAI (0.141 and 0.831, respectively [118,119]). However, coastal birds on the mainland are harder to buffer as urban areas often support large source populations of non-native predators (e.g. rodents) that can repeatedly reinvade bird habitat, even after eradication efforts [120]. Nest site height has also been shown to be a reliable predictor of urban tolerance in passerine-focused studies [10,16,36,41,50,51,58,59]. Our study supplements these previous findings to further reveal that nest site height is related to urban tolerance across a broad swath of the avian tree.

Approximately one-third of the species in our analysis exhibit nest site flexibility, using both high and low nest sites. This behaviour was observed frequently in several families that were well-represented in our dataset (i.e. had more than 50 species) including 28% of gulls and terns (family Laridae, 21 out of 74 species), 43% of ducks and geese (family Anatidae, 54 out of 126 species) and 51% of rails and coots (family Rallidae, 25 out of 49 species). When we removed these species from our analysis, the positive relationship between urban tolerance and nest site height was even stronger, which suggests that species with nest site flexibility did not drive our finding of nest site height as a predictor of urban tolerance. However, the abundance of mixed nest strategies in coastal bird species provides an interesting opportunity for future research and highlights the need to consider how intraspecific variability in functional traits relates to intraspecific variability in urban tolerance. Dark-eyed juncos (*Junco hyemalis*), a ubiquitous songbird in western North America, nests on the ground in its natural habitat; yet, in an urban population in San Diego, CA, off-ground nests are frequently observed and are associated with increased reproductive success [121]. Similar studies focused on coastal species with flexible nesting strategies that quantify the prevalence of high versus low nest sites in relation to the degree of urbanization are needed. Specifically, studies that determine whether reproductive success for a species varies with nest site height across a rural-to-urban gradient could provide valuable insight into how nest height impacts avian urban tolerance.

We also found that life-history traits are important predictors of urban tolerance in coastal birds. Coastal species that have more breeding attempts across their lifetime (lower brood value) produce larger clutches and have altricial young all exhibit higher urban tolerance. These strategies could be beneficial in urbanized environments where novel disturbances, exposure to pollutants and introduced predators can disrupt the success of nesting birds. Casting a ‘wide net’ by having larger clutches or having more frequent breeding events could help subsidize the inevitable loss of young in urban environments. Previous studies on passerines have found a positive relationship between larger clutches and urban tolerance [10,16,37,51,54] (but see [9,55]), and we detected the same pattern in coastal birds.

We predicted that coastal species that produce precocial young would do better in urbanized areas because they are more developed at hatching, require less parental care and thus may be better adapted to deal with disturbance. Two past studies that have examined primarily passerines have not detected a relationship between developmental mode and urban tolerance [9,40]. Interestingly, our results are opposite of our prediction; species with altricial young, such as yellow-crowned night herons (*Nyctanassa violacea*) and double-crested cormorants (*Phalacrocorax auritus*), are associated with higher urban tolerance using the MUTI and UN indexes, which is consistent with findings reported by [16]. It may be beneficial in urban environments for coastal birds to minimize the nesting stage, which is a characteristic of altricial species. Additionally, species that allocate less energy towards a clutch’s incubation may be able to produce more clutches per year [122], thus lowering their brood value, which was also supported by our results as a strategy for increased urban tolerance.

Coastal species that never defend a territory, with the exception of the small area around their nest site, were more urban tolerant than those that are seasonally or weakly territorial, a finding that was also supported by [9,10]. Defending a territory may be impractical in urban areas where habitat patches are often fragmented and resources are differentially distributed than in natural landscapes [3]. Gregarious species that naturally coexist in groups may be better suited to tolerate urban areas. For example, anthropogenic noise causes birds to spend more time scanning for predators and less time foraging [123,124]. However, species that live in groups may be able to counteract some of the negative effects that noisy conditions have on foraging and vigilance through ‘safety in numbers’ [125]. Gulls are social, non-territorial coastal birds that are frequently observed in urban areas around the world. They were abundant in our data and probably contributed considerably to the finding of higher urban tolerance in non-territorial species; in total, 34 out of 41 gull species represented by the UAI index have higher than average urban tolerance, including slaty-backed gull (*Larus schistisagus*; UAI = 2.92), Belcher’s gull (*Larus belcheri*; UAI = 2.55) and Audouin’s gull (*Ichthyaetus audouinii*; UAI = 2.58).

Many previous passerine-focused studies have found that diet is an important determinant of urban tolerance [9,10,37,40,41,51], and our results indicate that diet may also influence urban tolerance in coastal birds. Insectivorous birds have been frequently identified as urban avoiders [37,41] (but see [50]), probably due to reduced insect abundance in urbanized areas [126]. Similarly, our analysis finds that coastal birds with invertebrate diets are negatively associated with urban tolerance, supported by the MUTI index. However, our ‘invertebrate’ category includes not only insects but also encompasses aquatic and marine invertebrates such as molluscs, bivalves and crustaceans. Benthic coastal invertebrate communities have experienced decreased abundance and diversity due to urbanization [127,128]. Coastal invertivores may be less urban tolerant as a group due to this decreased abundance of both insect and aquatic invertebrate prey sources. For example, Seaside sparrows (*Ammospiza maritima*) have a low MUTI score (−1.372) and a diet that consists of both insects and marine invertebrates. We also found a negative trend between diets high in plants and seeds and urban tolerance of coastal birds using the index UN. Some prior evidence from passerines supports a negative relationship between granivorous diets and urban tolerance [16,37,51] (but see [9,41]). Reduced urban tolerance in species with plant/seed diets may be due to lower native plant diversity in urban areas [129].

The lack of relationship between peak vocal frequency and urban tolerance in coastal birds aligns with our initial hypothesis. Higher peak vocal frequencies have been repeatedly linked with increased urban tolerance in birds as these species probably experience less masking of their vocalization by low-frequency anthropogenic noise [39,46–48] (but see [49]). We expected that peak vocal frequency would not be an important indicator of urban tolerance in coastal birds, as they may already have higher vocal frequencies to avoid masking from the low-pitched background noise of the ocean [130]. However, a recent global analysis that incorporated species from nearly every family and order of birds actually found lower frequency vocalizations in coastal birds, possibly to help reduce attenuation in relatively open coastal habitats [131]. More targeted studies have found contrasting results, with species that live closer to the ocean singing higher-frequency songs [132]. Furthermore, multiple species of songbirds adjust their vocalizations in the presence of surf sounds [133,134]. Clearly the link between vocal frequency and coastal habitats, and how they relate to the urban tolerance of these species, warrants further investigation as selection for higher vocal frequencies may be strongest in species that live directly adjacent to the ocean, who must constantly overcome masking by surf sounds.

Although we did not find a relationship between dim light vision and urban tolerance in coastal birds, artificial light at night (ALAN) has emerged as a sensory pollutant that has important ecological effects on coastal birds. ALAN has been shown to disorient fledgling seabirds, such as Cory’s shearwaters (*Calonectris borealis*) [135], and has led to increased mortalities for a number of petrel and shearwater species (order Procellariiformes), with species that rear their young in underground nests seeming to be particularly vulnerable [136]. Additionally, a growing body of work shows that artificial illumination can alter foraging behaviour and predator detection in wading shorebirds, including dunlin (*Calidris alpina*), common ringed plover (*Charadrius hiaticula*) and Eurasian curlew (*Numenius arquata*) [137,138]. It is possible that our lack of finding between dim light vision and urban tolerance is due to a modest sample size of species-specific dim light vision measures (CT ratios) across all indexes. Expanding the number of species that have CT ratios could provide essential insights into how coastal birds will respond to our increasingly bright world.

In summary, we found that urban-tolerant coastal species nest high above the ground and have fast life-history strategies that include large clutch sizes, altricial young and relatively low brood values. Notably, UAI, the most species-rich index, indicated that the subset of coastal birds have higher average urban tolerance compared with the entire list of species, a finding that was also documented in [37]. Moving forward, it will be important to expand existing urban tolerance indexes to encompass more coastal species, so that we may gain a clearer understanding about the differences in urban tolerance between species occupying coastal and inland areas. Indexes that capture intraspecific variability in urban tolerance across a species’ range, such as UAI [10], are particularly promising as they can provide valuable insights about which functional traits confer urban tolerance in different biomes (e.g. tropical versus temperate areas). Furthermore, as many species within this group undergo long-distance migrations, using stop-over sites that may fall within urban areas, it will also be important to consider the timing of when various coastal species interact with urban environments. Finally, assessing which functional traits are unique indicators of urban tolerance in coastal bird species in the context of coastal-specific urbanization constraints could help inform conservation and natural resource management in the future, as development along coastlines is only projected to increase.

## Data Availability

All data files and code to reproduce the results presented in this manuscript are available through Zenodo [139]. Supplementary material is available online [140].
